# Moral judgement by the disconnected left and right cerebral hemispheres: a split-brain investigation

**DOI:** 10.1098/rsos.170172

**Published:** 2017-07-26

**Authors:** Conor M. Steckler, J. Kiley Hamlin, Michael B. Miller, Danielle King, Alan Kingstone

**Affiliations:** 1 Department of Psychology, University of British Columbia, 2136 West Mall, Vancouver, British Columbia, Canada V6T 1Z4; 2 Department of Psychological and Brain Sciences, University of California Santa Barbara, Santa Barbara, CA, USA; 3 School of Behavioral and Brain Sciences, University of Texas at Dallas, Richardson, TX, USA

**Keywords:** moral judgement, split-brain, intent-based evaluation

## Abstract

Owing to the hemispheric isolation resulting from a severed corpus callosum, research on split-brain patients can help elucidate the brain regions necessary and sufficient for moral judgement. Notably, typically developing adults heavily weight the intentions underlying others' moral actions, placing greater importance on valenced intentions versus outcomes when assigning praise and blame. Prioritization of intent in moral judgements may depend on neural activity in the right hemisphere's temporoparietal junction, an area implicated in reasoning about mental states. To date, split-brain research has found that the right hemisphere is necessary for intent-based moral judgement. When testing the left hemisphere using linguistically based moral vignettes, split-brain patients evaluate actions based on outcomes, not intentions. Because the right hemisphere has limited language ability relative to the left, and morality paradigms to date have involved significant linguistic demands, it is currently unknown whether the right hemisphere alone generates intent-based judgements. Here we use nonlinguistic morality plays with split-brain patient J.W. to examine the moral judgements of the disconnected right hemisphere, demonstrating a clear focus on intent. This finding indicates that the right hemisphere is not only necessary but also sufficient for intent-based moral judgement, advancing research into the neural systems supporting the moral sense.

## 1. Introduction

Typically, *intent* holds a privileged role in moral judgements [1–4]. Individuals tend to evaluate intentional harms as more blameworthy and wrong than injuries that were caused accidentally. Indeed, even if no negative outcome occurs, people will hold individuals responsible if they mean to cause harm [5]. Thus, a standard indicator of developmentally mature moral judgement is the extent to which the judgement takes the actor's intent into account [6].

Recent brain imaging and lesion studies have sought to reveal the brain regions underlying intent-based moral judgements. Consistent with the preferential lateralization of language to the left hemisphere, moral judgement tasks in which moral vignettes are presented linguistically have revealed that various regions of the left hemisphere seem to be involved in intent-based moral judgement, including but not limited to the left medial prefrontal cortex, left temporal parietal junction and left cingulate [4,7–9].

However, it is the right temporoparietal junction (rTPJ) that appears to be critically involved in processing mental state information such as intention and belief [4,10]. Indeed, recent work demonstrates that activity in the rTPJ is not only associated with intent-based moral judgement but also necessary for it [11,12]. Specifically, transcranial magnetic stimulation (TMS)-induced disruption of rTPJ activity increases reliance on outcomes in typically developing participants' assignment of blame to individuals in moral vignettes [11]. Also consistent with this conclusion, a recent moral evaluation study involving split-brain patients—for whom communication between the hemispheres is disconnected due to surgical severing of the corpus callosum—demonstrated that the disconnected left hemisphere generates moral judgements based primarily on outcomes, regardless of the acting individual's intent [13]. These findings suggest that when isolated from the rTPJ, the left hemisphere's intent-based moral judgements are profoundly compromised. Interestingly, the rTPJ seems to depend critically on upstream input from the right frontal system, as partial anterior callosotomy patients are also unable to produce intent-based moral judgements [13].

Together, these data demonstrate that the right hemisphere plays a critical role in left hemisphere intent-based moral judgement. However, because tasks used to date have typically involved fairly complex linguistic demands, it is not known if the (relatively) language impoverished right hemisphere can perform intent-based moral judgements on its own, independent of the language proficient left hemisphere. A recent set of fMRI studies reporting left lateralized activation for immoral linguistic *and* pictorial stimuli suggests that the right hemisphere alone may not be sufficient for moral evaluation [14]; however, this study did not consider intent-based moral judgements. Therefore, right hemisphere sufficiency for intent-based judgement remains a possibility.

An investigation that uses a callosotomy patient should shed light on the question of whether the right hemisphere is sufficient for intent-based moral judgement, because in split-brain patients the two hemispheres are cortically disconnected, allowing for the role of one hemisphere to be studied independently from the other. However, because the right hemisphere is relatively linguistically impoverished [15–17], traditional linguistic-vignette moral judgement tasks are inappropriate for examining moral judgements in the isolated right hemisphere. Thus, in the current study we explored intent-based moral judgement using a set of nonverbal ‘morality plays’; for example, videos depicting agents who intend to help versus hinder another agent's unfulfilled goal. These plays were originally designed to test the sociomoral evaluations of preverbal infants, and generate reliable evaluations in this preverbal population [18–20]. Critically, the use of these same non-linguistic moral stimuli with a split-brain patient allowed us to assess the intent-based moral judgements of the right hemisphere independently from the left. Furthermore, our non-linguistic tasks allowed us to examine the performance of both the left and right hemispheres under the very same testing conditions. That is, unlike past work exploring the brain bases of intent-based moral judgements, in our task the left hemisphere did not receive input in a (linguistic) format that it is specialized for processing.

We tested the split-brain patient J.W. on multiple sessions over a period of 1 year. Each session consisted of J.W. viewing several videos of entirely nonverbal morality plays; in each there was a protagonist with some unfulfilled goal, and two agents who acted to facilitate or block the protagonist's goal (see electronic supplementary material for details). J.W. saw two main types of morality plays during each session: (i) basic and (ii) intent-specific. In basic morality plays, the relative moral value of the agents was distinguishable both by their intentions and by the outcomes they caused; for example, one agent intentionally helped the protagonist (e.g. opening a box and therefore allowing the protagonist to get an object) and the other agent intentionally hindered the protagonist (e.g. blocking the box from being opened thereby preventing the protagonist from getting the object inside). In these cases, J.W. could successfully distinguish the characters by focusing solely on outcome (e.g. whether the protagonist got the object or not), entirely ignoring intent. These basic plays were included primarily as warm-ups to get J.W. used to the procedure at the start of each session, and also to provide a short intermission between various intent-specific morality plays.

In the intent-specific plays, the relative moral value of the two agents was *only* distinguishable by intention; this distinction occurred in one of two ways. First, the agents might hold different intentions toward the protagonist, yet bring about the exact same outcome (positive or negative). An example of this is a scenario whereby an agent with antisocial intentions deliberately prevented the protagonist from achieving its goal to put a toy on a high shelf by purposefully knocking the shelf over, and another agent with neutral intentions accidentally prevented the protagonist from achieving its goal by clumsily knocking the shelf over. Here, although the agents' intentions differed, the protagonist always experienced a negative outcome. Second, agents might hold different intentions toward the protagonist, and each be associated with an outcome opposite to what they had intended. An example of this type included an agent with positive intentions trying but failing to help the protagonist open a box to retrieve a toy (resulting in a negative outcome: no toy for the protagonist), and another agent with negative intentions trying but failing to hinder the protagonist in opening the box (resulting in the positive outcome: toy for the protagonist). These two types of intent-specific scenarios were of primary interest.

For every scenario, J.W. watched each agent alternately act on the protagonist two times each, for a total of four events. Then, J.W. was presented with pictures of the two agents, one depicted on the top and one depicted on the bottom of a blank white screen. He was then asked ‘who is nicer?’ (note that even the linguistically impoverished right hemisphere is capable of processing very simple linguistic instructions such as this). In response to the prompt, J.W. indicated his selection by pointing to the top or bottom character using either his left index finger (a movement initiated by the right hemisphere) or his right index finger (initiated by the left hemisphere) [21]. By requiring J.W. to use a contralateral finger to provide a response, we were able to isolate responding to one hemisphere at a time. One hemisphere was tested at a time for an entire block, which consisted of both basic and intent-specific scenarios (see methods for details). We examined whether each hemisphere evaluated prosocial agents as nicer than antisocial agents—on both basic and intent-specific plays—beyond random chance of 50%.

## 2. Results and discussion

**Figure 1.**
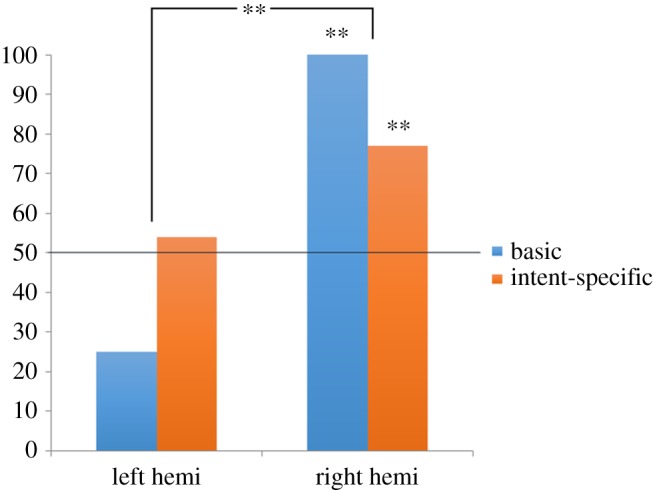
The *Y*-axis represents the percentage of choices for the relatively nicer agent. The horizontal line across the 50% mark represents chance responding. For both hemispheres, responses to basic events are shown in blue bars and to intent-specific events in orange bars. Overall (i.e. combined basic and intent-specific) performance of the left and right hemispheres differed. The right hemisphere demonstrated typical moral judgement (evaluating more prosocial agents as nicer) on both basic and intent-specific morality plays whereas the left hemisphere failed to distinguish the characters beyond chance levels for both event types. ** signifies statistical significance at *p* < 0.01.

There was a significant difference in overall (i.e. combined basic and intent-specific) performance on the moral judgement task between the left and right hemispheres, Fisher's exact test, *p* = 0.005 (figure 1). Breaking this result down, J.W.'s right hemisphere selected as ‘nicer’ the relatively more prosocial agents both in the basic shows (8/8 correct, binomial test *p* = 0.008) and in the intent-specific shows (20/26 correct, binomial test *p* = 0.009). In contrast, J.W.'s left hemisphere chose randomly on both the basic shows (2 out of 8 correct, binomial test *p* = 0.29) and the intent-specific shows (14/26 correct, binomial test *p* = 0.85). For intent-specific shows, J.W.'s responding (for either hemisphere) did not depend on whether the valence of the outcome for the protagonist was positive or negative, or whether the show depicted a failed attempt or an accident (*p*s > 0.30).

Our results indicate that the disconnected right hemisphere is capable of generating adult-typical intent-based moral judgements, evaluating agents with positive intentions as nicer than those with negative intentions. This finding is consistent with prior work showing a critical role for the right hemisphere in generating intent-based moral judgements, perhaps via the operation of the rTPJ [11]. By contrast, the left hemisphere did not solve either basic or mentalistic moral contrasts. Our findings are the first to suggest that the combined workings of circuits in both cerebral hemispheres is not necessary for generating intent-based moral judgements. Rather, our results suggest that the right hemisphere is necessary and sufficient, and the left hemisphere is not necessary, for generating intent-based moral judgements.

One possible explanation for this result is that the right hemisphere—and not the left—was merely responding to the valence associated with each agent. From this perspective, the right hemisphere may not necessarily be engaging in moral judgement at all, but rather some lower-level valence evaluation. We view this possibility as unlikely, given that valenced outcomes are arguably *more* salient than are valenced intentions—particularly in the case of accidental acts. Prior work has supported this possibility: people under cognitive load prioritize outcomes, not intentions, in their moral judgements, suggestive that outcomes are more salient/easily processed than intentions are [22]. Results suggest that J.W. did not respond based on outcome. Thus, if the right hemisphere were merely tracking valence, it would probably not have succeeded.

One fascinating question from our results is why J.W.'s left hemisphere performed at chance levels even on the basic events, when agents were distinguishable based on outcome alone. Indeed, previous studies using linguistic stimuli have found that the left hemisphere makes reliable moral judgements based on outcome [13]. Critically however, in previous linguistic tasks subjects were unambiguously asked for their moral judgements (e.g. by being asked whether an action was morally acceptable or unacceptable) [13]. In contrast, the present investigation used the question ‘Who is nicer?’. Critically, ‘niceness’ does not solely refer to the moral dimension. Therefore, our design left it up to each hemisphere as to whether it would interpret the question as a moral-based query or not. Our results suggest that in this ambiguous situation, the left hemisphere does not default to a moral assessment, whereas the right hemisphere does.

Anecdotal evidence from our study supports this interpretation. For example, when J.W.'s left hemisphere was asked *why* he chose a particular agent on one trial, his left hemisphere said that girls with blonde hair cannot be trusted, apparently ignoring the behaviour the agent performed (one agent happened to have blonde hair in this scenario). On another trial, J.W.'s left hemisphere said that he chose as he did because the box moved the most during that [antisocial] trial. Of course, it is difficult to say whether these linguistic reasons caused the left hemisphere judgements or were merely *ex post facto* verbal rationalizations for judgements made for other reasons [23,24]. However, they do dovetail with previous split-brain studies indicating a propensity for the left hemisphere to create false hypotheses in order to explain events, even when no obvious patterns exist. For example, in a study using a probability guessing game in which a dot randomly flashes in one location 80% of the time and another location 20% of the time, the left hemisphere failed to note the overall probability of dot flashes and instead tried to figure out the ‘pattern’ at which dots appeared, though none existed. In contrast, in the same study the right hemisphere did not search for specific patterns, and instead learned to maximize by repeatedly selecting the more probable location [25].

In conclusion, our study has demonstrated that the right hemisphere, when cortically isolated from the left hemisphere, is sufficient for making intent-based moral judgements. The present results also raise the interesting possibility that new insights may be garnered using stimuli that leave ambiguous whether a judgement task is moral. Indeed, in the real-world people presumably make moral judgements spontaneously, as opposed to being instructed to do so as in most experimental work to date. The present results suggest that the left hemisphere, when isolated from the right, may not default to a moral perspective.

## 3. Method

Videos and pictures of the agents were presented using Microsoft PowerPoint on a Macbook computer resting on a table. J.W. watched the videos sitting in a chair about an arm's length away from the screen. There were two basic split-brain methodology options for testing J.W. One option was to present the stimulus to one hemisphere at a time, by flashing visual information briefly in either the left or right visual fields. The other was to present the information to both hemispheres at once, but then to probe the left or right hemisphere independently at the time of response by obtaining responses from the hand contralateral to the hemisphere being tested. Because the stimulus displays were extended videos, lateralization of the input to the left or right visual field was not feasible; thus we opted for the latter methodology*.* This methodological approach is quite standard in split-brain research and one where both hemispheres have commonly performed successfully [26]. In addition, although our study used a single split-brain patient, J.W. is representative of the select population of split-brain patients that have been studied in the past [26].

Each block consisted of different basic and intent-specific morality plays (11 total per block), with two basic plays coming at the beginning as a warm-up to the task (not included in analysis) and two or three basic plays interspersed among six or seven intent-specific shows (number varying by session). Rare trials in which one hemisphere tried to respond out of turn, or attempted to interfere with the response of the hemisphere being tested, were excluded from analysis. Because we wanted to ensure that J.W. had processed each event, he saw each agent act on the protagonist's goal twice in alternation before being asked to identify which agent was nicer using his contralateral index finger. An artificial red curtain was used to separate the events. Note that on the last round of testing J.W. saw each agent act on the protagonist's goal once, rather than twice, in alternation, as he was now familiar with the general design. The data from this session was comparable to those of the previous sessions.

The morality plays consisted of a combination of video recorded live-action puppets moving around on a black stage, filmed in a developmental psychology laboratory, as well as animated shows created in Microsoft PowerPoint (see electronic supplementary material for details). Both across sessions and within morality plays, several factors were counterbalanced to keep J.W. from possibly associating certain traits (e.g. the colour of a puppet's shirt) with the niceness or meanness of an agent. The identity of the agents was counterbalanced across show type and session number (e.g. whether the nicer agent wore a green shirt or a blue shirt), as was the order in which a particular agent acted (e.g. the nicer agent sometimes acted first or second), what side of the protagonist they appeared on (left or right), and whether they were presented to J.W. on the top or bottom of a screen during testing (i.e. during the ‘who is nicer’ question).

## Supplementary Material

Nonverbal Morality Plays

## Data accessibility

The dataset supporting this manuscript is deposited at the Open Science Framework: https://osf.io/q9kvb/.
