# Supplementary material for: Moral judgement by the disconnected left and right cerebral hemispheres: a split-brain investigation
Source: R Soc Open Sci. 2017 Jul 26;4(7):170172. doi: 10.1098/rsos.170172 (PMC5541538; doi:10.1098/rsos.170172)
Supplement: Nonverbal Morality Plays [file rsos170172supp1.docx]

**Supplemental Online Material**

S1. **Morality Plays and Descriptions.**

We utilized several different nonverbal morality plays (to view the shows, see: <http://cic.psych.ubc.ca/media/steckler-et-al-stimuli/>).

**Basic Shows**

*Box Show*: In this show, a puppet (the “Protagonist”) tried, with difficulty, to open the lid of a clear plastic box with a brightly-colored object inside. The Protagonist was alternately aided by a prosocial puppet (the “Helper”), who grabbed the side of the box and helped to open it, and thwarted by an antisocial puppet (the “Hinderer”) who slammed the box lid closed, preventing the Protagonist from getting the toy (see Hamlin & Wynn, 2011).

*Ball Show*: In this show, a Protagonist played with a ball at the middle of a puppet stage, repeatedly jumping up and down and tossing and catching the ball. The Protagonist then lost its ball, which bounced to one side of the stage. The ball was retrieved by one of two puppets that rested at the back corners of the stage area. One puppet (the “Giver”) gave the ball back to the Protagonist, while another puppet (the “Taker”) ran offstage with the ball (see Hamlin & Wynn, 2011).

*Hill Show*: In this show, a Protagonist repeatedly attempted but failed to climb up a steep hill. On the third climb attempt, the Protagonist was either pushed up the hill by one puppet (the “Helper”), or pushed down by another puppet (the “Hinderer”; see Hamlin, Wynn, & Bloom, 2007).

*Shelf Show*: In this show, a toy sat on top of one side of a wooden box. A Protagonist tried, but failed, to jump high enough to reach the toy on top of the box. The Protagonist was alternately aided by a prosocial puppet (the “Helper”), who leapt on top of the box, grabbed the toy, and dropped it down to the Protagonist, or was thwarted by an antisocial puppet (the “Hinderer”), who leapt on top of the box, grabbed the toy, and ran off stage with it.

*Stack Show*: In this show, a blue square platform lay in the center of the stage next to three blue circular shaped toys. In alternation, one of two events occurred. In one event, one Protagonist motioned back and forth between the platform and the toys and then attempted but failed to stack the toys of the platform. On the Protagonist’s last attempt to lift the toys, a prosocial puppet (the “Helper”) aided the Protagonist by helping to lift and stack the toys on the platform. In the other event, a different Protagonist motioned back and forth between the platform and the toys and successfully stacked the toys on the platform. After the toys were stacked, an antisocial puppet (the “Hinderer”) ran up to the stacked toys and knocked them over.

**Intent-Specific Shows**

*Accidental Helping*: In this show, a toy sat on top of one side of a clear box; a second toy sat on the ground near the front of the box. After checking out the toy on the ground, a Protagonist tried, but failed, to reach the preferred toy on top of the box. The Protagonist was alternately aided by one puppet (the “Intentional Helper”), who ran up to the box and intentionally pushed it over so that the Protagonist could get the toy, or was “aided” by another puppet (the “Accidental Helper”), who, running toward the toy on the ground, happened to knock the shelf over allowing the Protagonist to get the toy.

*Accidental Hindering*: In this show, a toy sat on the ground of one side of a clear box; a second toy sat on the ground near the front of the box. After checking out the toy on the ground, a Protagonist tried, with difficulty, to place a toy on top of the box; the Protagonist eventually succeeded. This goal was then thwarted by one puppet (the “Intentional Hinderer”), who ran up to the box and intentionally pushed it over so that the Protagonist’s toy was no longer atop the box, or was “thwarted” by a puppet (the “Accidental Hinderer”), who, running for the toy on the ground, happened to knock it over reversing the Protagonist’s goal.

*Failed Attempts, Both Agents Fail*: In this show, a puppet (the “Protagonist”) tried, but failed, to open the lid of a clear plastic box with a brightly-colored object inside. The Protagonist was alternately acted on by a prosocial puppet (the “Failed Helper”), who grabbed the side of the box and tried to help open it but couldn’t, and by an antisocial puppet (the “Failed Hinderer”), who tried to slam the box lid closed but ultimately the Protagonist got the box open anyway (see Hamlin, 2013). In this show, the “nicer” agent is the one who had good intentions (to help) but is associated with a bad outcome; the “meaner” agent had bad intentions but is associated with a good outcome.

*Failed Attempts, Successful Helper, Failed Hinderer*: In this show, a puppet (the “Protagonist”) tried, but failed, to open the lid of a clear plastic box with a brightly-colored object inside. The Protagonist was alternately acted on by a prosocial puppet (the “Successful Helper”), who grabbed the side of the box and helped to open it, and by an antisocial puppet (the “Failed Hinderer”), who tried to slam the box lid closed but ultimately the Protagonist got the box open anyway (see Hamlin, 2013). In this show, the “nicer” agent is the one who had good intentions (to help) and did so, despite that both agents’ actions were associated with the same good outcome (the Protagonist getting the toy).

*Failed Attempts, Failed Helper, Successful Hinderer*: In this show, a puppet (the “Protagonist”) tried, but failed, to open the lid of a clear plastic box with a brightly-colored object inside. The Protagonist was alternately acted on by a prosocial puppet (the “Failed Helper”), who grabbed the box and tried to help open it but couldn’t, and by an antisocial puppet (the “Successful Hinderer”), who slammed the box lid closed, preventing the Protagonist from getting the toy (see Hamlin, 2013). In this show, the “nicer” agent is the one who had good intentions (to help) but both agents’ actions were associated with the same bad outcome (the Protagonist not getting the toy).

*Rock Hill*: In this show, a Protagonist rest at the bottom of a hill before repeatedly attempting to climb up it. On the third climb attempt, the Protagonist was prevented from climbing the hill. The Protagonist was either pushed down the hill by a large rock that was intentionally pushed down the hill by an antisocial puppet, or, the Protagonist was pushed down the hill by a rock whose falling was just associated with a puppet who happened to be touching the rock as the hill shook violently, causing the rock to fall (show modified from Hamlin, Wynn, & Bloom, 2007).

*Wall Show*: In this show, a large pillar or wall sat in the middle of the screen. In the Intentional Hinderer case, the Intentional Hinderer was on one side of the wall while the Protagonist was on the opposite side of the wall. The Intentional Hinderer moved to the other side and looked intently up at the Protagonist. The Intentional Hinderer then moved back in place, again on opposite sides of the wall to the Protagonist, and began to run up and push the wall, in an attempt to knock it over on top of the Protagonist. On the third attempt, the Intentional Hinderer successfully pushes the wall over on top of the Protagonist, who becomes squished and who displays a sad face. In the Accidental Hinderer case, the show is identical except that the Protagonist isn’t on stage for much of the show. When the Accidental Hinderer moves around to look on the other side of the wall, the space is empty. The Accidental Hinderer looks upwards before moving back to other side of the wall. The Accidental Hinderer then ran up and pushed the wall, in an attempt to knock it over, and with no reason to assume this act would harm anyone. On the third attempt, the Protagonist entered from the left of the screen (i.e., in a different direction from where the Accidental Hinderer gazed when on that side of the wall) and was squished by the wall as it fell over, again displaying a sad face.
